# Image quality at low tube voltage (70 kV) and sinogram-affirmed iterative reconstruction for computed tomography in infants with congenital heart disease

**DOI:** 10.1007/s00247-015-3372-2

**Published:** 2015-06-27

**Authors:** Motoo Nakagawa, Yoshiyuki Ozawa, Keita Sakurai, Masashi Shimohira, Kazuya Ohashi, Miki Asano, Sachiko Yamaguchi, Yuta Shibamoto

**Affiliations:** Department of Radiology, Nagoya City University Graduate School of Medical Sciences, 1 Kawasumi, Mizuho-cho, Mizuho-ku, Nagoya, Japan; Division of Central Radiology, Nagoya City University Hospital, Nagoya, Japan; Department of Cardiovascular Surgery, Nagoya City University Graduate School of Medical Sciences, Nagoya, Japan; Department of Pediatrics and Neonatology, Nagoya City University Graduate School of Medical Sciences, Nagoya, Japan

**Keywords:** Congenital heart disease, Dual-source CT, Sinogram-affirmed iterative reconstruction, Infant

## Abstract

**Background:**

Lower tube voltage has advantages for CT angiography, such as improved contrast

**Objective:**

To evaluate the image quality of low-voltage (70 kV) CT for congenital heart disease and the ability of sinogram-affirmed iterative reconstruction to improve image quality.

**Materials and methods:**

Forty-six children with congenital heart disease (median age: 109 days) were examined using dual-source CT. Scans were performed at 80 kV and 70 kV in 21 and 25 children, respectively. A nonionic iodinated contrast medium (300 mg I/ml) was used for the 80-kV protocol. The contrast medium was diluted to 75% (225 mgI/mL) with saline for the 70-kV protocol. Image noise was measured in the two protocols for each group by extracting the standard deviations of a region of interest placed on the descending aorta. We then determined whether sinogram-affirmed iterative reconstruction reduced the image noise at 70 kV.

**Results:**

There was more noise at 70 kV than at 80 kV (29 ± 12 vs 20 ± 4.8; *P* < 0.01). Sinogram-affirmed iterative reconstruction with grade 4 strength settings improved the noise (20 ± 5.9; *P* < 0.01) for the 70-kV group.

**Conclusion:**

Sinogram-affirmed iterative reconstruction improved the image quality of CT in congenital heart disease.

## Introduction

Due to recent technological advances in computed tomography (CT), the morphological features of vessels and cardiac chambers in children with congenital heart disease can be noninvasively evaluated in a short time [1]. Three-dimensional CT angiography is typically performed with low tube voltages in children because the attenuation of contrast media becomes higher. Three-dimensional imaging methods such as volume rendering and maximum intensity projection can easily be reconstructed if the contrast enhancement of vessels also increases in children. Moreover, the amount of contrast media can be reduced by using a lower tube voltage [2, 3].

While the usefulness of an 80-kilovoltage (kV) protocol has been reported in contrast-enhanced CT for congenital heart disease [4, 5], the second generation of dual-source 128-slice CT systems (Somatom Definition Flash, Forchheim, Germany) can generate even lower voltage (70 kV) X-rays. However, image noise may increase on CT scans using a lower tube voltage [6]. Therefore, minimizing the radiation dose while maintaining acceptable image quality has remained a challenge in clinical practice. Iterative reconstruction methods have recently been proposed to solve this problem, and could reduce image noise and maintain image quality for CT scans at a lower dose. The recently introduced sinogram-affirmed iterative reconstruction is the latest advance in the field of iterative reconstruction [7]. The purpose of our study was to compare the image quality of 80-kV and 70-kV CT protocols and evaluate the effects of sinogram-affirmed iterative reconstruction on the image quality of low-dose (70 kV) CT for congenital heart disease.

## Materials and methods

The Institutional Review Board approved this retrospective study. The requirement for written informed consent was waived for the retrospective analysis.

### Patients

Forty-six children with congenital heart disease (ages 1 day to 1 year; median: 109 days; body weight: 2.1 to 8.0 kg; median: 3.5 kg) were examined using dual-source CT between April 2013 and January 2014. The types of congenital heart disease were an atrial septal defect in 2 patients, ventricular septal defect in 16, tetralogy of Fallot in 12, patent ductus arteriosus in 7, atrioventricular septal defect in 1, single ventricle in 3, double-outlet right ventricle in 4, interruption of the aortic arch in 2, hypoplastic left heart syndrome in 2, persistent truncus arteriosus in 1 and corrected transposition of the great arteries in 1. Five children each had two defects. No limit was set for heart rate. Exclusion criteria were impaired renal function and parental refusal. The present study required image analysis; therefore, all procedures were performed within the confines of clinical practice. The potential risks of radiation exposure and the contrast medium injection were explained to all parents by a pediatric cardiologist. Written informed consent was obtained before CT. Clinical management and decisions as well as the children’s privacy and rights were not compromised.

### Cardiac multidetector computed tomography protocol

All children were examined by a second-generation dual-source CT system (Somatom Definition Flash; Siemens Healthcare, Forchheim, Germany). The acquisition collimation was set to 128 × 0.6 mm. Scans were performed with a prospectively ECG-triggered high-pitch (3.4) spiral acquisition (FLASH Spiral Cardio; Siemens Healthcare, Forchheim, Germany). CT scans were performed at 80 kV and 70 kV in 21 and 25 children, respectively. The tube current was adjusted to the child’s characteristics using automodulation (CARE Dose 4D; Siemens Healthcare, Forchheim, Germany) with quality reference mAs/rotation set to 210 mAs and reference kV set to 120 kV.

A 300 mg I/ml nonionic contrast medium (Omnipaque300 iohexol; Daiichi-Sankyo, Tokyo, Japan, or Iopamiron300 iopadmidol; Bayer Schering Pharma, Berlin, Germany) was used for the 80-kV protocol. Because lower kV increases iodine contrast [8], the contrast medium was diluted with saline to 75% (225 mg I/ml) for the 70-kV protocol. The contrast medium was injected through the dorsum manus or dorsal foot vein at a volume of 2 ml/kg body weight. A saline chaser was not used. The flow rate was set at body weight × 0.1 ml/s for children with a body weight >5 kg. Scanning was started just after completion of the contrast injection. A fixed flow rate of 0.5 ml/s was used for children whose body weight was ≤5 kg. Bolus tracking was used to synchronize the contrast medium injection with scanning by applying the region of interest within the descending aorta at the level of the carina. After the CT number reached 150 Hounsfield units, a 2-s post-threshold delay was set before the scan. The scan was performed from the supraclavicular level to the lowest end of the lung without breath holding.

### CT image reconstruction

**Fig. 1:**
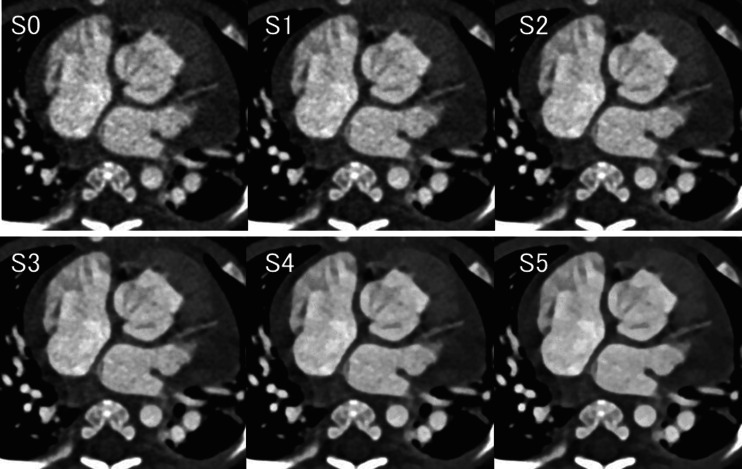
Contrast-enhanced CT transverse images in a 2-month-old boy. This six-image series was reconstructed for each child in the 70-kV group, including one data set reconstructed by a conventional filtered back projection algorithm (S0) and five data sets reconstructed by sinogram-affirmed iterative reconstruction with five different (increasing) strengths (S1-5)

Six image serials were reconstructed for each child in the 70- and 80- kV groups, including one data set reconstructed by a conventional filtered back projection algorithm (S0) and five data sets reconstructed by sinogram-affirmed iterative reconstruction with five different strength settings (S1-5) (Fig. 1). All data sets were reconstructed with a slice thickness of 0.75 mm. Each child’s weight was measured and recorded just before the CT examination.

### Objective image evaluation

#### Comparison of image noise between the 70-kV and 80-kV protocols

A region of interest was placed on the descending aorta. The CT number (Hounsfield unit) and image noise were objectively measured for 70-kV and 80-kV image data sets reconstructed by a conventional filtered back projection algorithm (S0). The region of interest in the descending aorta was >8 mm^2^. Image noise was measured by extracting the standard deviations of the CT number of the region of interest. Artifacts such as beam hardening were carefully excluded from region of interest measurements of the descending aorta. CT number and image noise were determined as the average of three independent measurements. We then compared these data between the 70- and 80-kV protocols.

We also evaluated the contrast-to-noise ratio. The contrast-to-noise ratio was calculated as (mean attenuation of descending aorta - mean attenuation of erector spine muscle) divided by image noise within muscle [9]. The muscle region of interests were all >10 mm^2^.

#### Evaluation of sinogram-affirmed iterative reconstruction for image quality of low-dose CT

We investigated whether sinogram-affirmed iterative reconstruction could reduce the image noise of 70-kV image data. We measured the noise within the descending aorta for image data reconstructed by the filtered back projection algorithm (S0) and sinogram-affirmed iterative reconstruction (S1-5), and compared this to the results obtained with the 80-kV protocol reconstructed by the filtered back projection algorithm.

### Subjective image evaluation

Two board-certified radiologists (M.N. and Y.O. with 11 and 12 years of clinical experience, respectively), who have reviewed more than 300 adult and pediatric cardiac multidetector CT scans per year and were blinded to the image techniques, individually evaluated the images. Any discrepancies were resolved by consensus.

#### Evaluation of beam hardening artifacts

Contrast-enhanced CT with a low kV may produce beam hardening artifacts due to a dense contrast medium in the superior vena cava. We evaluated the degree of beam hardening artifacts in the 70- and 80-kV protocols. Cases in which the contrast medium was injected through the dorsal foot vein were excluded; therefore, 22 cases in the 70-kV group and 18 cases in the 80-kV group were evaluated. All of the reconstructed images were loaded into a CT workstation (Syngo MultiModality Workplace; Siemens, Erlangen, Germany). CT data sets were reconstructed by sinogram-affirmed iterative reconstruction with S5 for 70-kV and 80-kV protocol. The window level and width were freely changed to visualize CT images. The CT data sets were randomized and the readers were blinded to the acquisition parameters.

Beam hardening artifacts were evaluated with a 4-point scale as follows

**Fig. 2:**
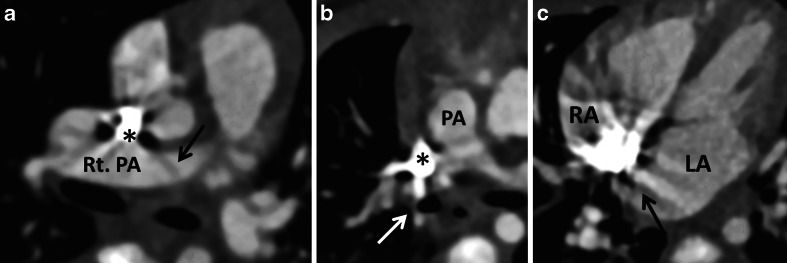
Example images of beam hardening artifacts due to a dense contrast medium in the superior vena cava (*). **a** Contrast-enhanced CT transverse images of a 26-day-old girl. A beam hardening artifact (Grade 2, *arrow*) may have affected the evaluation of the right pulmonary artery (Rt. PA). **b** Contrast-enhanced CT images of a 29-day-old boy. Beam hardening (Grade 3, *arrow*) made it hard to evaluate the pulmonary artery (PA). **c** Contrast-enhanced CT transverse images of a 3-month-old boy. A beam hardening artifact (Grade 3, *arrow*) disturbed the depiction of the atrium septum. *LA* left atrium, *RA* right atrium

- Grade 0: There was no beam hardening artifact.
- Grade 1: A beam hardening artifact existed but did not affect the image evaluation.
- Grade 2: A beam hardening artifact existed and could affect the evaluation of structures around the superior vena cava, such as the aorta, pulmonary artery, atrium septum, and Blalock-Taussig shunt.
- Grade 3: Beam hardening was too hard to evaluate the morphological features of the structures around the superior vena cava. Example images of Grade 2 and 3 are shown in Fig. 2.

#### Subjective evaluation of image quality of the pulmonary artery

**Fig. 3:**
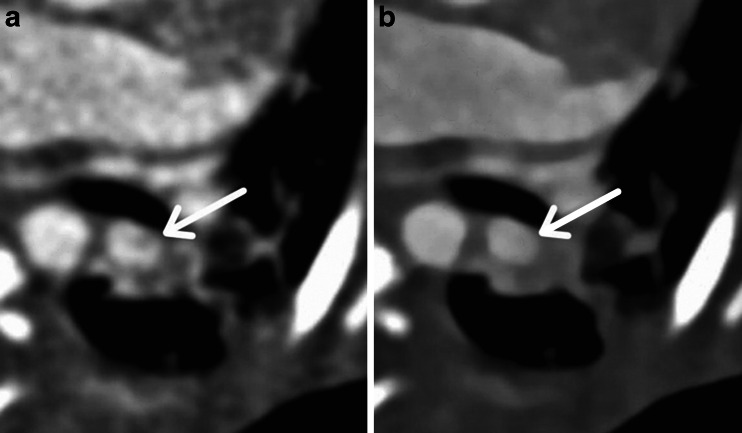
Contrast-enhanced CT transverse images of a 35-day-old girl. Example images of image noise in the left pulmonary artery. **a** CT image of 70-kV (S0, pure filtered back projection). There was image noise (Grade 2) in the left pulmonary artery (*arrow*), which might be confused with embolism. **b** CT image of 70-kV (S5, high-strength sinogram-affirmed iterative reconstruction). There was no image noise in the left pulmonary artery (*arrow*)

Thrombosis of pulmonary artery, shunt or conduit can be problems for children with congenital heart disease. When we interpret cardiac CT images that are scanned with low-kV protocol, image noise in vessels and shunt tube is sometimes confused with embolism, which shapes linear or band-like configurations (Fig. 3) [10]. In this study, we evaluated how the image noises confuse our image interpretation. CT data sets were reconstructed by sinogram-affirmed iterative reconstruction with S0 and S5 for 70-kV protocols and S0 for 80-kV protocol. We evaluated image noise of the pulmonary artery of left lower lobe with a 4-point scale as follows.

- Grade 0: There was no image noise.
- Grade 1: There was image noise, but we did not confuse it as embolism.
- Grade 2: There was image noise, and we could confuse it with embolism.
- Grade 3: There was image noise, and we could not differentiate it from embolism at all.

We chose the pulmonary artery of the left lower lobe for evaluating image noise because this artery was unlikely to be affected by beam hardening artifacts from the superior vena cava. Example images of Grade 0 and Grade 2 are shown in Fig. 3. In this study, we excluded cases that might have a possibility of embolism. Specifically, cases whose D-dimer was more than 1.0 μg/ml in laboratory tests done 21 days before CT scan were excluded. D-dimer was examined by latex photometric immunoassay method. Twenty-two cases in the 70-kV group and 18 cases in the 80-kV group were evaluated.

#### Radiation dose

We compared mean dose-length product of groups of 80 kV and 70 kV. With the dose-length product displayed on the CT system (phantom size is 32 cm), the effective dose for each child was calculated by using the following equation: E = k × (dose-length product). K is 0.0823 and 0.0525 mSv mGy-1 cm-1, which is a conversion coefficient for the chest at 80 kV in the newborn and 1 year old, respectively [11]. Size-specific dose estimates of 80 kV and 70 kV were also calculated [12]. Anterior-posterior and lateral diameters were measured at transverse CT image at level of carina. For the sum of these diameters in each child, conversion factors were chosen from the table of the Report of AAPM Task Group 204 (http://www.aapm.org/pubs/reports/rpt_204.pdf) for 32-cm phantom size. Then, size-specific dose estimates were calculated as follows: Size-specific dose estimates = computed tomography dose index × conversion factor (mGy).

### Statistical analysis

The Shapiro-Wilk W test was used to determine whether image noise, attenuation and degree of beam hardening were normally distributed. Parametric data were compared using the paired *t*-test, and nonparametric data were tested with the Wilcoxon signed rank test.

For subjective evaluation, interobserver agreement was calculated by using kappa statistics. Kappa scores of 0.41–0.60, 0.61–0.80 and greater than 0.80 were regarded to be indicative of moderate, good and excellent agreement, respectively [13].

## Results

### Objective comparisons

Children and CT acquisition characteristics are summarized in Table 1. CT numbers of the region of interest at the descending aorta were 490 ± 130 and 500 ± 120 with the 80- and 70-kV protocols, respectively, and were not significantly different (*P* = 0.69). Image noises reconstructed with the filtered back projection algorithm were 21 ± 4.8 and 29 ± 13 with the 80- and 70-kV protocols, respectively (*P* = 0.0020). Image noise in the 70-kV protocol was higher than that in the 80-kV protocol. Dose-length product and effective dose of 80-kV and 70-kV protocols are also summarized in Table 1. Seventy-kV protocol could reduce the radiation exposure.

**Table 1:** Patient and scan data (±standard deviation) with the 80-kV and 70-kV protocols

	80 kV	70 kV	*P*-value
Number of children	21 (6 neonates)	25 (7 neonates)	
Body weight (kg)	3.9 ± 1.7	4.1 ± 1.8	0.55
CT number of the aorta (HU)	490 ± 130	500 ± 120	0.69
Image noise of data set reconstructed by conventional filtered back projection	21 ± 4.8	29 ± 13	0.0020
CT dose index (mGy)	0.98 ± 0.46	0.52 ± 0.14	0.00024
Size-specific dose estimate (mGy)	2.4 ± 1.1	1.4 ± 0.34	0.00050
Dose length product (mGy cm)	17 ± 9.1	9.9 ± 3.7	0.0010
Effective dose (mSv)	1.1 ± 0.61	0.58 ± 0.18	0.0016

**Fig. 4:**
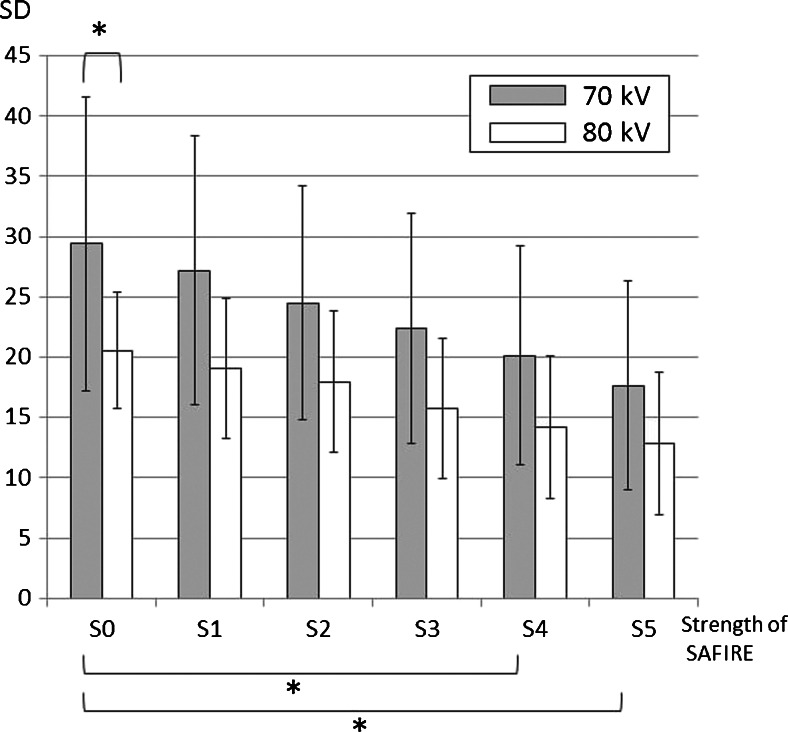
Image noise in the 70-kV and 80-kV protocols. A significant difference was observed between the two protocols in the noise at S0 (pure filtered back projection). In the 70-kV protocol reconstructed by filtered back projection (S0) and sinogram-affirmed iterative reconstruction (S1-5), significant differences were noted between S0 and S4-5. * *P* < 0.01 *SAFIRE* sinogram-affirmed iterative reconstruction SD standard deviation

**Fig. 5:**
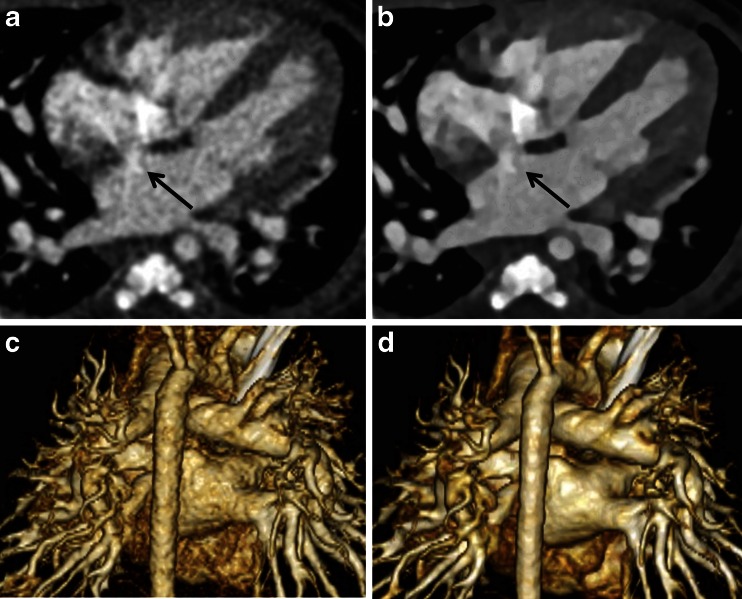
A 1-month-old girl with a ventricular septal defect and atrium septal defect (*arrows* in **a** and **b**). CT was scanned using the 70-kV protocol. **a**-**b** Transverse images are reconstructed by a conventional filtered back projection algorithm (S0) and sinogram-affirmed iterative reconstruction with the 5th strength (S5), respectively. **c**-**d** Volume-rendered images reconstructed by S0 and S5, respectively

**Fig. 6:**
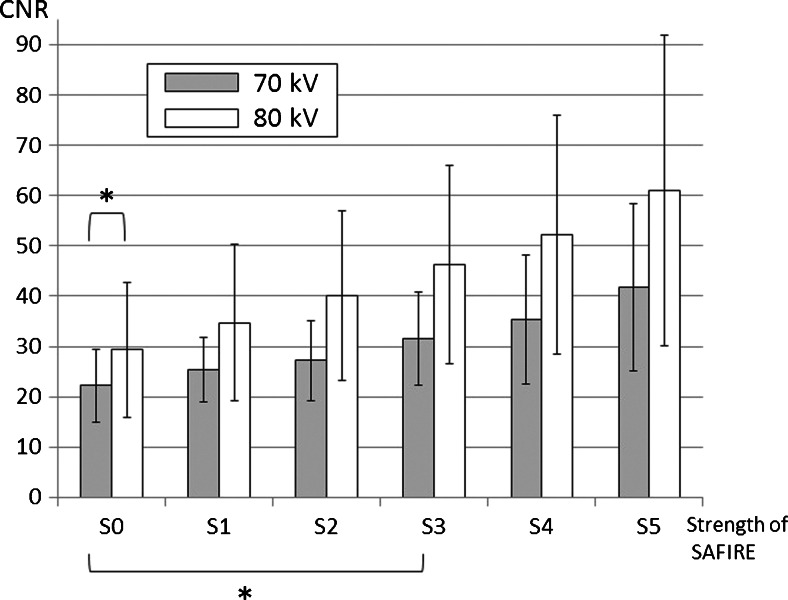
The contrast-to-noise ratio of 70-kV and 80-kV protocols for the data set with S0-5. A significant difference was observed between the contrast-to-noise ratio of S0 and S3-5 (*P* < 0.01) in the 70-kV protocol. There was no difference between contrast-to-noise ratios of the 80-kV protocol (S0) and that of S1-4 of the 70-kV protocol (*P* > 0.1). *: *P* < 0.01, SAFIRE: sinogram-affirmed iterative reconstruction, CNR: contrast-to-noise ratio

The measured objective image noise of 70 kV for the data set with filtered back projection (S0) and the 5 data sets with sinogram-affirmed iterative reconstruction (S1-5) were (mean ± S.D.) 29 ± 12, 27 ± 11, 25 ± 9.7, 22 ± 9.5, 20 ± 9.1, and 18 ± 8.7, respectively (Fig. 4). The noise at 80 kV for the data set with filtered back projection (S0) and the 5 data sets with sinogram-affirmed iterative reconstruction (S1–5) were 20 ± 4.8, 19 ± 5.8, 18 ± 5.9, 22 ± 5.8, 20 ± 5.9, and 18 ± 5.9, respectively (Fig. 4). Transverse CT images and volume rendering images of example cases for the 70-kV protocols are shown in Fig. 5. A significant difference was observed between the noise at S0 and S4-5 (*P* < 0.01) in the 70-kV protocol. The image noise level of 70 kV with S4 was the same as that of 80 kV with S0. In addition, the contrast-to-noise ratios of 70 kV for the data set with S0-5 were 22 ± 7.3, 25 ± 6.5, 27 ± 7.9, 32 ± 9.2, 35 ± 13 and 42 ± 17, respectively (Fig. 6). The contrast-to-noise ratios at 80 kV for the data set with S0-5 were 29 ± 14, 35 ± 16, 40 ± 17, 46 ± 20, 52 ± 24 and 61 ± 31, respectively (Fig. 6). A significant difference was observed between the contrast-to-noise ratio of S0 and S3-5 (*P* < 0.01) in the 70-kV protocol. There was no difference between contrast-to-noise ratios of 80-kV protocol and S1-4 of 70-kV protocol (*P* > 0.1).

### Subjective comparisons

The grades of beam hardening artifacts are shown in Table 2. Beam hardening artifacts were slightly stronger in the 70-kV protocol than in the 80-kV protocol (*P* = 0.083). Interobserver agreement was excellent (Kappa = 0.86). There were five cases of Grade 3 artifacts in the 70-kV protocol group; three cases in the pulmonary arteries and two in the atrial septum (Table 2, Fig. 2).

**Table 2:** Grade of beam hardening artifacts

	80 kV (*n* = 18)	70 kV (*n* = 22)	*P*-value
Grade 0	2	0	-
Grade 1	4	5	-
Grade 2	12	12	-
Grade 3	0	5	-
Average grade	1.6 ± 0.70	2.0 ± 0.69	0.076

The grades of image quality of pulmonary arteries are shown in Table 3. Average of score of 80 kV (S0), 70 kV (S0) and 70 kV (S5) were 0.95 ± 0.69, 1.5 ± 0.51 and 0.45 ± 0.51, respectively. Image noise in pulmonary artery was fewer in 70-kV (S0) protocol than in 80-kV (S0) and 70-kV protocols (*P* = 0.017 and *P* < 0.001). Interobserver agreement was excellent (Kappa = 0.81). There were 4 and 10 cases of Grade 2 artifacts in the 80-kV (S0) and 70 kV (S0) protocol group, respectively (Table 3, Fig. 3).

**Table 3:** Grade for image quality of pulmonary artery at 80 kV and 70 kV. S0 signifies image reconstruction with filtered back projection. S5 is reconstruction with the highest factor of sinogram-affirmed iterative reconstruction. The grading is significantly different between 80 kV and 70 kV with both S0 and S5 (P < 0.05), and between S0 and S5 at 70 kV (*P* < 0.001)

	80 kV (*n* = 18)	70 kV (*n* = 22)	
	S0	S0	S5
Grade 0	5	0	12
Grade 1	11	12	10
Grade 2	4	10	0
Grade 3	0	0	0
Average grade	0.95 ± 0.69	1.5 ± 0.51	0.45 ± 0.51

## Discussion

The attenuation of contrast media becomes higher at a lower voltage and the image contrast increases [7]. Several previous studies have shown that the contrast medium dose could be reduced when a low tube voltage technique was used [2, 3]. In our study, no significant difference was observed in the CT number of CT angiography between the 80-kV protocol using a 300 mgI/mL contrast medium and the 70-kV protocol with a diluted contrast medium (225 mgI/mL). Our study revealed that the amount of contrast medium could be reduced with the lower tube voltage (70 kV) protocol in CT angiography for infants with congenital heart disease.

The most commonly used dose of nonionic contrast media appears to be 2 ml/kg (300 mgI/ml) [14]. One of the major disadvantages of using an iodine-based contrast medium is the risk of renal dysfunction. Niboshi et al. [15] reported renal dysfunction in children with cardiovascular disease following cardiac angiography using a nonionic contrast medium. Although renal tubular function was intact on a long-term basis, they suggested that care was needed to prevent contrast media-induced nephropathy, especially in neonates, infants and children receiving more than 5 ml/kg of a contrast medium in total. Based on the findings of our study and previous ones, a dose of 2 ml/kg appears to be relatively safe in children with intact renal function [14]. However, because children with congenital heart disease may need to be repeatedly examined with cardiac catheterization or CT angiography, reducing the total amount of contrast media is desired.

Image noise may increase on CT scans using a lower tube voltage [6]. Our study revealed that image noise in the 70-kV protocol was higher than that in the 80-kV protocol due to lower radiation dose of the 70-kV protocol. Because the CT number can be raised with a lower tube voltage protocol, such a protocol can make volume rendering images easier to create. However, high image noise may degenerate volume rendering images [16] (Fig. 5). Therefore, the ability to achieve acceptable image quality remains a challenge in clinical practice.

Iterative reconstruction techniques have been proposed for more than three decades to improve CT image quality by reducing quantum noises and artifacts; however, computer power has only recently increased enough to provide clinically acceptable reconstruction times [17]. These iterative reconstruction algorithms use multiple reconstruction iterations to reduce image noises and artifacts. Sinogram-affirmed iterative reconstruction has a unique feature that uses two iteration reconstruction loops: one iterated in the raw data domain to reduce image artifacts and the other in the image domain to reduce noise [7]. Previous studies showed that sinogram-affirmed iterative reconstruction could maintain image quality with a significant reduction in the radiation dose for coronary and body CT angiography [7, 17, 18]. We confirmed that sinogram-affirmed iterative reconstruction was also useful for pediatric CT angiography to reduce image noise objectively. Moreover, we demonstrated the ability of five reconstruction strengths of sinogram-affirmed iterative reconstruction to reduce image noise. S4 could improve the image noise level of the 70-kV protocol more than that of the 80-kV protocol with the filtered back projection algorithm (S0). The objective image quality is not necessarily related to visual image quality [7]. In this study, we also evaluated image quality subjectively. Image noises of 80- or 70-kV protocols (S0) sometimes could confuse radiologists (average grade as 0.95 ± 0.69 and 1.5 ± 0.51, respectively). We confirmed that sinogram-affirmed iterative reconstruction can improve CT images of low-kV protocol to reduce the image noise that radiologists confuse as embolism (Table 3). This suggests clinical utility of the technique (Fig. 3).

X-rays passing through an object become “harder,” i.e. the mean energy increases, because lower-energy photons are absorbed more rapidly than higher-energy photons. This phenomenon causes so-called beam hardening artifacts [19]. The use of a lower kV protocol is more likely to cause beam hardening artifacts. Although no significant difference was observed in beam hardening artifacts between the 80- and 70-kV protocols in our study (Table 2), we consider that the quality of CT images with a low kV protocol could be decreased by beam hardening artifacts (Fig. 2). Further studies are warranted on this issue. For example, diluting the contrast medium can be regarded as one method. Artifices such as keeping the child’s hands up and removing medical devices such as a monitoring lead and nasogastric tube may also be effective.

The 70-kV protocols further reduces the radiation exposure compared to the 80-kV technique (Table 1). This may be beneficial for repeated imaging in certain conditions that require stepwise correction: pulmonary artery banding, Blalock-Taussig shunt and Glenn and Fontan operation. Although coronary artery anomaly sometimes makes intracardiac repair difficult [20], coronary arteries of infants are occasionally hard to depict clearly due to their small configuration [21]. Additional CT angiography for estimation of coronary artery may be needed in conditions such as tetralogy of Fallot after growth and before intracardiac repair. Therefore, low kV protocol with sinogram-affirmed iterative reconstruction is the ideal technique for CT angiography to examine morphological features of congenital heart disease because of lower radiation dose.

### Study limitation

We have referred data of the displayed report on dual-source CT (Somatom Definition Flash; Siemens Healthcare, Forchheim, Germany) using 32-cm phantom. Therefore, effective doses of our data must have been estimated lower than those of a 16-cm phantom, which is more adaptable for children [22]. However, size-specific dose estimates of our data should be accurate because we used the conversion factor for a 32-cm phantom for estimating size-specific dose estimates [12].

Deak et al. [11] reported that conversion factors decrease with increases in voltage. Therefore, the conversion factor may be different between 70 kV and 80 kV. However, we had to use the conversion factor for 80 kV proposed by Deak et al. [11] since the conversion factor of 70 kV were not available.

## Conclusion

A low tube voltage (70 kV) technique may be adapted for CT angiography in infants with congenital heart disease. Sinogram-affirmed iterative reconstruction improved image quality in the low tube voltage protocol in our study.
